# Interorgan Communication Between Lung and Colorectal Epithelial Cells Studied Using a Novel Multi‐Organ‐On‐Chip System

**DOI:** 10.1002/cph4.70051

**Published:** 2025-09-15

**Authors:** Brady Rae, Verena Bood, Hye‐Jin Dijk, Gwenda F. Vasse, Barbro N. Melgert, Anika Nagelkerke, Janette K. Burgess, Dirk‐Jan Slebos, Irene H. Heijink, Simon D. Pouwels

**Affiliations:** ^1^ Department of Pathology and Medical Biology University Medical Center Groningen, University of Groningen Groningen the Netherlands; ^2^ GRIAC Research Institute University Medical Center Groningen Groningen the Netherlands; ^3^ Department of Molecular Pharmacology Groningen Research Institute for Pharmacy, University of Groningen Groningen the Netherlands; ^4^ Department of Pharmaceutical Analysis Groningen Research Institute for Pharmacy, University of Groningen Groningen the Netherlands; ^5^ Department of Pulmonary Diseases Groningen Research Institute for Pharmacy, University of Groningen Groningen the Netherlands

**Keywords:** COPD, interorgan communication, lung‐gut axis, microplastics, multi‐organ‐on‐chip

## Abstract

Chronic Obstructive Pulmonary Disease (COPD), a severe lung disease caused by chronic inhalation of toxic gases and particles, is often accompanied by extrapulmonary comorbidities. These are characterized by systemic inflammation and activation of the bi‐directional lung‐gut axis, in which communication takes place between lung and intestinal cells. The mechanisms of interorgan communication in COPD are largely unknown, partly due to the lack of suitable in vitro models to study interorgan communication. In the current study, we developed a novel unidirectional millifluidic multi‐organ‐on‐chip (MOoC) device, in which stimulated lung epithelial cells were connected to colorectal cells. Human lung epithelial A549 cells were exposed to cigarette smoke extract and nylon microplastic fibers, mimicking inhaled pollutants that induce lung epithelial damage and can contribute to the development of COPD. Once exposed, A549 cells were connected to naïve colorectal DLD‐1 cells within our MOoC system to study interorgan communication mediated by released factors such as cytokines, chemokines, or Damage Associated Molecular Patterns (DAMPs). A549 cells treated with inhalable pollutants released communication mediators, such as the DAMP galectin‐3. Naïve DLD‐1 cells responded to these released factors from stimulated A549 cells by inducing pro‐inflammatory responses, demonstrated by increased *IL‐6* mRNA expression and decreasing barrier integrity, as demonstrated by decreased *CDH1* mRNA expression and delocalization from the cell membrane of E‐cadherin and ZO‐1 proteins. This study introduces a novel chip platform that can be used to study communication between cells derived from different organs. This study also provides relevant insight into the mediators involved in lung–gut axis communication.

## Graphical abstract

In this study, a new in vitro model has been developed that can be used to study interorgan communication via released factors. This unidirectional millifluidic multi‐organ‐on‐chip (MOoC) device was used to study the communication between lung and colorectal cells.

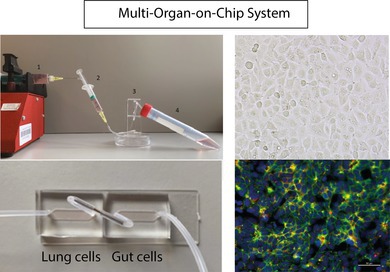

## 1 Introduction

Chronic Obstructive Pulmonary Disease (COPD) is characterized by inflammation of the airways (chronic bronchitis) and destruction of alveolar tissue (emphysema). COPD is caused by the recurrent inhalation of toxic gases and particles, of which cigarette smoke is the most well‐known example. However, exposure to other pollutants such as exhaust fumes, particulate matter, or microplastics can also damage the epithelial layer and contribute to the development of COPD (Paplińska‐Goryca et al. 2025).

In recent years, it has become evident that COPD patients experience systemic effects that affect not only the lungs but also other organs throughout the body. Several studies have shown that over half of all COPD patients experience co‐morbidities such as cardiovascular diseases, osteoporosis, or metabolic syndrome, and that the prevalence of these comorbidities is higher than may be expected based on age, smoking, or social‐economic status (Santos et al. 2022; Divo et al. 2012; Fabbri et al. 2023). Additionally, it was shown that COPD patients have an increased risk for the development of gastrointestinal diseases, such as inflammatory bowel disease and gastric ulcers (Labarca et al. 2019; Vutcovici et al. 2016). Many of these diseases have overlap in the factors driving their susceptibility and onset, increasing the chance of developing multi‐morbidity. However, it has been postulated previously that the lungs can communicate with other organs via inflammatory and cellular damage‐related factors that can spill over from the lungs of COPD patients into the circulation, triggering extrapulmonary effects (Barnes and Celli 2009). Nevertheless, in the following years, it appeared difficult to gather substantial evidence for this so‐called “spill‐over” hypothesis (Roca et al. 2014; Miller et al. 2013). To date, limited efforts have been reported to perform functional studies on interorgan communication in relation to the onset and development of extra‐pulmonary manifestations of COPD. Of note, most of the studies investigating the spill‐over hypothesis were cohort studies, mainly focusing on pro‐inflammatory cytokines (Barnes and Celli 2009; Nowiński et al. 2019; Brassington et al. 2019; Decramer and Janssens 2013).

Organs can communicate with each other using factors released from cells that end up in the bloodstream, after which they can eventually activate and trigger responses in cells from other organs. These secreted factors are interorgan communication mediators, such as cytokines, chemokines, microRNAs, extracellular vesicles, and damage associated molecular patterns (DAMPs) (Tokizane and Imai 2024; Huang et al. 2024). In COPD, it has been shown that lung epithelial cells can secrete large amounts of cytokines, DAMPs, and extracellular vesicles upon exposure to inhalable pollutants such as cigarette smoke, exhaust fumes, or microplastics, (Eckhardt et al. 2022; Sharma et al. 2024; Pouwels et al. 2014; Song, van Dijk, et al. 2024) and that these factors can enter the bloodstream (Eckhardt et al. 2025; Pouwels et al. 2017; Brajer‐Luftmann et al. 2019; Hailong et al. 2024; Roodenburg et al. 2024).

The best studied example of interorgan communication in COPD is the lung‐gut axis. It has previously been shown that COPD patients have increased intestinal permeability and an altered gut microbiota (Liu et al. 2025). Multiple studies have shown that orally administered factors, such as pre‐ and probiotics, can improve COPD symptoms by inducing gut‐lung interorgan communication (Liu et al. 2025; Shen et al. 2024; Song, Meng, et al. 2024). Additionally, it was shown that tobacco smoking as well as exposure to air pollution can alter the gut microbiota composition and gut function (Otake et al. 2025). Together, these findings suggest bi‐directional communication between the lungs and the gut (Wang et al. 2023). While it has been shown that direct exposure of colorectal cells to cigarette smoke extract (CSE) reduces colorectal cell viability and barrier function, insight into cell‐derived factors from the lungs that affect the gut after cigarette smoking is missing (Dino et al. 2019). In order to better understand the cellular mechanisms of interorgan communication between the lungs and the gut, more sophisticated in vitro models in which cells from both organs can be cultured simultaneously and their communication can be studied are needed.

In the current study, we have developed a novel Multi‐Organ‐on‐Chip (MOoC) system to study interorgan communication between lung cells that were exposed to inhalable pollutants and colorectal cells. Several models connecting cells from different organs within a microfluidic device have been described previously (Marzagalli et al. 2022; Koning et al. 2021; Cook et al. 2025) However, these models are often not suitable to study the continuous communication between lung cells and colorectal cells, do not allow for timely connection and disconnection, or yield very small amounts of biological samples, such as supernatants, RNA, and cell lysates. Recently, we have developed a novel open‐source airway‐on‐chip model with a 100 mm^2^ cell growth area that can be used to grow large numbers of cells in limited amounts of medium under a constant flow, allowing the measurements of released factors (Rae et al. 2025) In the current study, this airway‐on‐chip model was redesigned to obtain a unidirectional millifluidic device connecting two 100 mm^2^ cell culture chips, optimized to model interorgan communication. In the current study, human lung epithelial A549 cells and human colorectal DLD‐1 cells were cultured in the MOoC system to study lung‐gut communication. In order to model epithelial damage induced by inhaled pollutants, A549 cells were exposed to CSE and nylon microplastic fibers, while the DLD‐1 cells were not exposed and remained naïve. Upon connection of the pollutant‐stimulated A549 cells and naïve DLD‐1 cells, a constant medium flow between these cells ensured the consistent exposure of DLD‐1 cells to factors released from A549 cells. Afterwards, inflammatory markers and barrier integrity were evaluated to study whether gut cells responded to these released factors.

## 2 Materials and Methods

### 2.1 Cell Culture and Exposures

Human alveolar carcinoma epithelial A549 cells and human adenocarcinoma colorectal epithelial DLD‐1 cells were acquired from American Type Culture Collection (ATCC). Both cell lines were cultured at 37°C with a humidified atmosphere containing 5% CO_2_ in RPMI‐1640 medium (Gibco) supplemented with 10% Fetal Bovine Serum (FBS) and 1% Penicillin–Streptomycin (P/S) (Invitrogen). Cells tested negative for the presence of mycoplasma before usage. CSE was prepared by bubbling smoke from two filterless 1R6F research cigarettes (Tobacco‐Health Research, University of Kentucky) through 25 mL of serum‐free RPMI‐1640 supplemented with 1% P/S, at a constant rate of 5 L/h using a peristaltic pump (Watson & Marlow; 603S). CSE was further diluted in serum‐free RPMI‐1640 to a concentration of 20% or 40%, after which it was used to stimulate cells within 30 min after preparation. A549 cells were stimulated with CSE at a constant flow of 150 μL/h for 4 h. A microplastic stock solution was prepared by diluting 6,6‐nylon textile fibers (AM325705; Goodfellow) with a median size of 12 × 31 μm in 1× PBS (Gibco) and 0.05% BSA to a 1 × 10^6^/mL concentration, as described previously (Song, van Dijk, et al. 2024). This stock solution was further diluted in RPMI‐1640 medium to a working concentration of 16.67 × 10^4^/mL, resulting in 10,000 fibers that exposed the A549 cells during the 4 h stimulation period at a constant flow of 150 μL/h. DLD‐1 cells were stimulated in the chip with 0, 10, or 50 μg/mL recombinant human Galectin‐3 for 24 h (Biolegend, Cat# 599706).

### 2.2 Multi‐Organ‐On‐Chip (MOoC) Assembly

**FIGURE 1:**
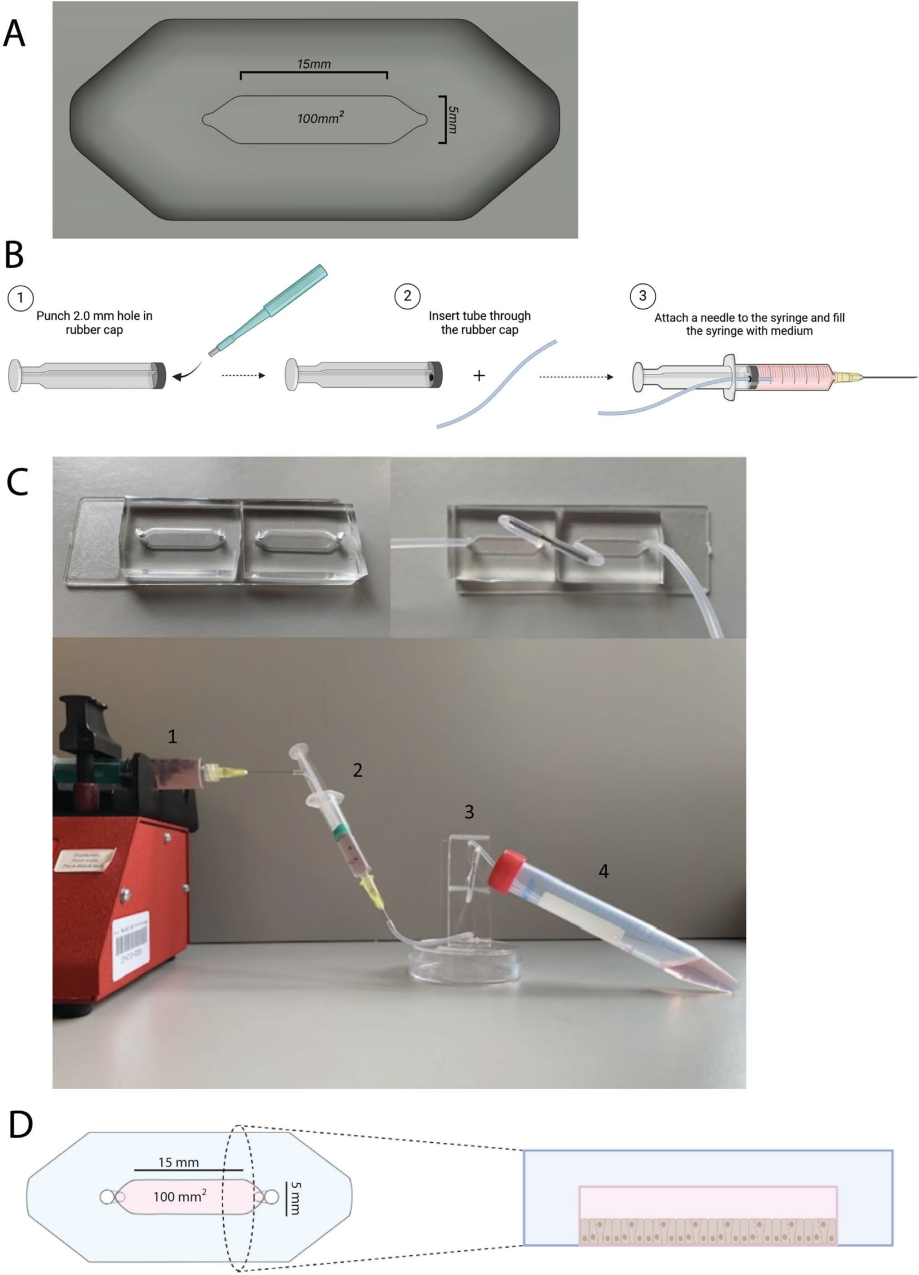
Design and construction of the Multi‐Organ‐on‐Chip (MOoC) system. (A) MOoC mold design. The cell culture chamber has a length of 15 mm (18 mm including the tapered area), a width of 5 mm, and a cell culture surface of 100 mm^2^. (B) Schematic representation of the construction of a bubble trap. A hole with a 2 mm diameter is punched in the rubber part of the 10 mL syringe plunger using a biopsy punch. Tubing can be slid through the 2 mm hole to form a tight fit. The bubble trap can be connected between the syringe pump and the MOoC chips using blunt needles. Created with biorender.com. (C) Photos illustrating the MOoC set‐up. The left upper panel depicts a top view of the cell culture chambers embedded on a polystyrene microscope slide. In the right upper panel, the culture chambers are connected using tubing and a blunt needle. The bottom photo depicts the complete MOoC set‐up with (1) a syringe filled with culture medium in a syringe pump, (2) connected to a syringebubble trap, (3) the bubble trap is connected to the vertically placed culture chambers mounted on a polystyrene microscope slide, and (4) the second culture chamber is connected to a collection tube, where outflow is collected and stored. (D) Schematic representation of the top and side view of the cell culture chamber. Cells grow on the bottom of the culture chamber, with growth medium flowing above the cells.

The design, shape, and size of the culture chambers used for the MOoC models were adapted from our recently in‐house developed open‐source millifluidic airway‐on‐chip model (Rae et al. 2025). The MOoC cell culture chambers have a 100 mm^2^ cell growth surface area and were prepared using a milled solid polymethyl methacrylate chip mold that was produced as described before (Figure 1A) (Rae et al. 2025). The height of the cell culture chamber is 1 mm, generating a medium volume of 100 μL in the chamber. To produce PDMS chips, 4 mL of a 1:10 weight ratio mixture of Silicone Elastomer Base and Silicone Elastomer Curing Agent (Sylgard 184, Dow Corning) was poured in the chip molds, followed by degassing the chips in a vacuum chamber for 30 min. Next, the chips were placed in a 65°C dry oven for 1.5 h to polymerize the PDMS. Afterwards, any excess PDMS was removed using a scalpel. Subsequently, in‐ and outlets were created using a 2.0 mm biopsy punch (Kai medical; BP‐80F) and connected using a Platinum Cured Silicone tube (0.5 mm × 0.8 mm (1/32); Darwin Microfluidics). Two cell culture chips were then attached to a 75 × 25 mm Nunc polystyrene microscope slide (Thermo Fisher Scientific) using liquid PDMS as glue. Lastly, the chips were incubated at 65°C for 1.5 h. To prevent the formation of air bubbles within the cell culture chambers, a bubble trap was placed between the syringe pump and the cell culture chamber. The bubble trap was constructed by punching a hole with a biopsy punch (2 mm) in the rubber cap of the plunger of a 2 mL syringe (BD, Becton, Dickinson and Company) (Figure 1B). Subsequently, both culture chambers were connected using two 1.75 cm long Platinum Cured Silicone tubes (0.5 mm × 0.8 mm diameter (1/32); Darwin Microfluidics) connected using a 0.9 mm needle (BD; Microlance‐3) to generate a connecting tube between the two cell culture chambers with a volume of approximately 7 μL. In order to assemble the whole MOoC model, a syringe pump (Prosense, PSNE1600) loaded with a 10 mL syringe was connected to the bubble trap and culture chambers using silicon tubing. The polystyrene microscope slide with the two culture chambers was placed vertically in a custom‐made holder to expel air bubbles upon formation. Lastly, the outlet of the second culture chamber was connected to a 15 mL collection container using silicon tubing to collect the outflow of the chip (Figure 1C,D).

### 2.3 Experimental Set‐Up

**FIGURE 2:**
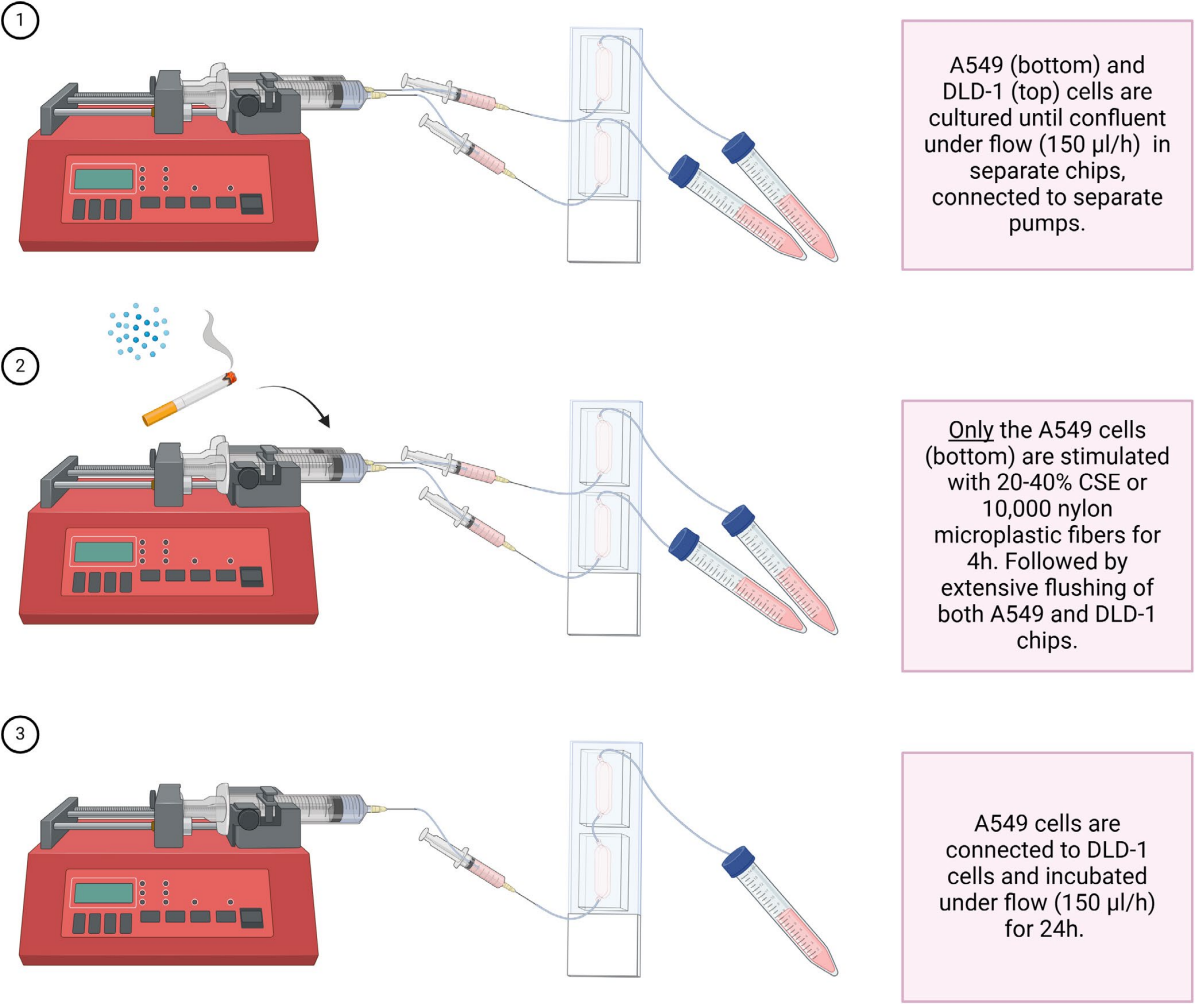
Experimental set‐up to study the communication between cigarette smoke extract (CSE) or microplastic‐exposed lung epithelial cells and colorectal epithelial cells. (1) Lung epithelial A549 cells and colorectal epithelial DLD‐1 cells are cultured in the Multi‐Organ‐on‐Chip (MOoC) system, on 150 μL/h flow using two separate pumps, until they reach confluency. (2) The A549 cells are stimulated with 20%–40% CSE, 10,000 nylon microplastic fibers, or control medium for 4 h. To match the conditions between cell lines, the DLD‐1 cells are exposed to control medium for 4 h. Afterwards, both culture chambers are flushed to remove any residual CSE or microplastic fibers from the A549 cells or as control for the DLD‐1 cells. (3) The A549 culture chamber is connected to the DLD‐1 culture chamber, followed by 24 h of incubation under 150 μL/h flow. Afterwards, cells are used for staining or for RNA isolation, and the outflow is frozen down until further usage. This figure was created with biorender.com.

In order to study the communication between CSE‐ or microplastic‐exposed lung epithelial cells and colorectal epithelial cells, A549 cells and DLD‐1 cells were cultured in the MOoC system. The first culture chamber was seeded with 50,000 A549 cells and the second culture chamber with 40,000 DLD‐1 cells. The cells were incubated for 24 h at 37°C and 5% CO_2_ under static conditions to allow cellular attachment. Afterwards, both cell culture chambers were connected to a separate syringe pump and outflow collection container (Figure 2), and incubated under 150 μL/h flow with RPMI‐1640 medium supplemented with 10% FBS and 1% P/S for 2 days, after which the cells had formed a confluent monolayer. Next, the A549 cells were stimulated with 0%, 20%, or 40% of CSE or 10,000 nylon microplastic fibers in serum‐free RPMI‐1640 with 1% P/S for 4 h under 150 μL/h flow, while the DLD‐1 cells were incubated for 4 h in serum‐free RPMI‐1640 with 1% P/S under 150 μL/h flow. Afterwards, both cell culture chambers were extensively flushed using 5 mL serum‐free RPMI‐1640 medium in order to remove any residual CSE or microplastics. Lastly, both cell culture chambers were connected to form the MOoC system, and the cells were incubated for 24 h under 150 μL/h flow of serum‐free RPMI‐1640 with 1% P/S.

### 2.4 Immunofluorescent Staining

Cells were fixed and permeabilized in the chip with methanol/acetone (1:1) for 30 min at room temperature. Afterwards, cells were washed three times with 1× PBS, subsequently blocked for 1 h with blocking solution (1% BSA in PBS; Sigma‐Aldrich) and incubated overnight with primary antibodies diluted in PBS at 4°C. See Table 1 for used antibodies and dilutions. Next, the cells were washed thrice with PBS and subsequently incubated with secondary antibodies for 1 h in the dark at room temperature. Afterwards, the cells were washed five times with PBS before adding DAPI (1:1000 in PBS: 1 mg/mL; Sigma Aldrich) for 10 min. The PDMS chip was removed from the polystyrene slide, and the cells were mounted with CitiFluor AF1 Mountant Solution (Electron Microscopy Sciences). Slides were imaged using the Leica DM4000b (Leica microsystems) microscope.

**TABLE 1:** Antibodies used for immunohistochemistry staining.

Antibody	Host	Dilution	Supplier
E‐cadherin	Rabbit polyclonal	1:100	Santa Cruz (sc‐7870)
ZO‐1	Mouse monoclonal	1:100	Invitrogen (33‐9100)
Alexa Fluor 647	Donkey anti‐mouse	1:500	Invitrogen (A31571)
Alexa Fluor 555	Donkey anti‐rabbit	1:500	Invitrogen (A31572)

To determine cell viability, cells were stained with Calcein AM 1:200 (1 mg/mL; BioLegend), Hoechst 1:50 (1 mg/mL; Thermo Fisher Scientific), and propidium iodide 1:100 (0.5 mg/mL; PI; Sigma‐Aldrich) in the chips for 30 min. Afterwards, the cells were washed once with Hank's Balanced Salt Solution (HBSS; Gibco). The PDMS cell culture chambers were removed from the polystyrene microscope slide and slides were air dried for 2–5 min before Neo‐Mount mounting medium (Sigma Aldrich) and a glass coverslip were applied. Cells were visualized using an EVOS FL fluorescent microscope (Life Technologies) using the GFP, TexasRed, and DAPI filters. All photos were taken with the same microscope settings.

Sub‐cellular staining analysis was done using the CellProfiler 4.2.8 software. To quantify the stainings at subcellular regions, objects were created for whole cell area, cell membrane area, and cytoplasm area (whole cell area minus cell membrane area and nucleus area). In short, the DAPI channel was used to detect the nucleus as objects; after rescaling the intensity of the DAPI image, nuclei objects were detected with the module IdentifyPrimaryObjects. Rescaling was done to ensure single nuclei were detected. To identify the whole cell as an object, the ECAD and ZO1 images were merged; this merged image was then used in the module identifySecondaryObjects using the nuclei as input objects. To create the cell membrane objects, the whole cell objects were shrunken by 3 pixels with the ExpandOrShrink module. These shrunken objects were then used in the IdentifyTertiaryObjects module, with the whole cell objects set as the larger objects and the shrunken objects set as the smaller objects, creating the cell membrane objects. Next, the module IdentifyTertiaryObjects was used again to create the cytoplasm objects by selecting the shrunken objects as the larger object and the nuclei objects as the smaller objects. With the modules MeasureObjectsSizeShape and MeasureObjectsIntensity, the total area and intensities were measured for each object for the ECAD and ZO1 staining separately.

### 2.5 Cellular Response Assays

Extracellular double‐stranded (ds)DNA levels were measured in the cell‐free outflow of the chip using the PicoGreen Fluorescent dsDNA Assay (Invitrogen) according to the manufacturer's protocol. The levels of galectin‐3 were measured using the Human Galectin‐3 DuoSet ELISA (DY1154; R&D Systems).

Gene expression levels were assessed by qRT‐PCR. Cells were lysed inside the chips with 100 μL TRIzol Reagent (Invitrogen). RNA was isolated following the user guide of TRIzol solution and subsequently quantified using the Nanodrop‐1000 (NanoDrop Technologies). Per sample, one microgram of RNA was converted to cDNA using the RevertAid First Strand cDNA Synthesis kit (Thermo Fisher Scientific). Gene expression was analyzed using the GoTaq Probe kit (Promega) and specific TaqMan primer‐probe sets (Thermo Fisher Scientific) for the following genes: *PPIA* (Hs99999904_m1), *CXCL8* (Hs00174103_m1), *IL‐6* (Hs00174131_m1), *CDH1* (Hs01023894_m1), and the ViiATM 7 Real‐Time PCR machine (Thermo Fisher Scientific). The expression of target genes was corrected for the expression of the house‐keeping gene (*PPIA*). The expression was plotted as the fold induction of the 2^−ΔCt^ values of the experimental conditions over the 2^−ΔCt^ of the control condition.

### 2.6 Statistics

Statistical differences between conditions were tested using a non‐parametric Mann–Whitney *U* test. *p* values < 0.05 were considered statistically significant. All data on which statistical analyses were done were performed as 6–7 independent experiments.

## 3 Results

### 3.1 Construction and Validation of the Multi‐Organ‐On‐Chip System

**FIGURE 3:**
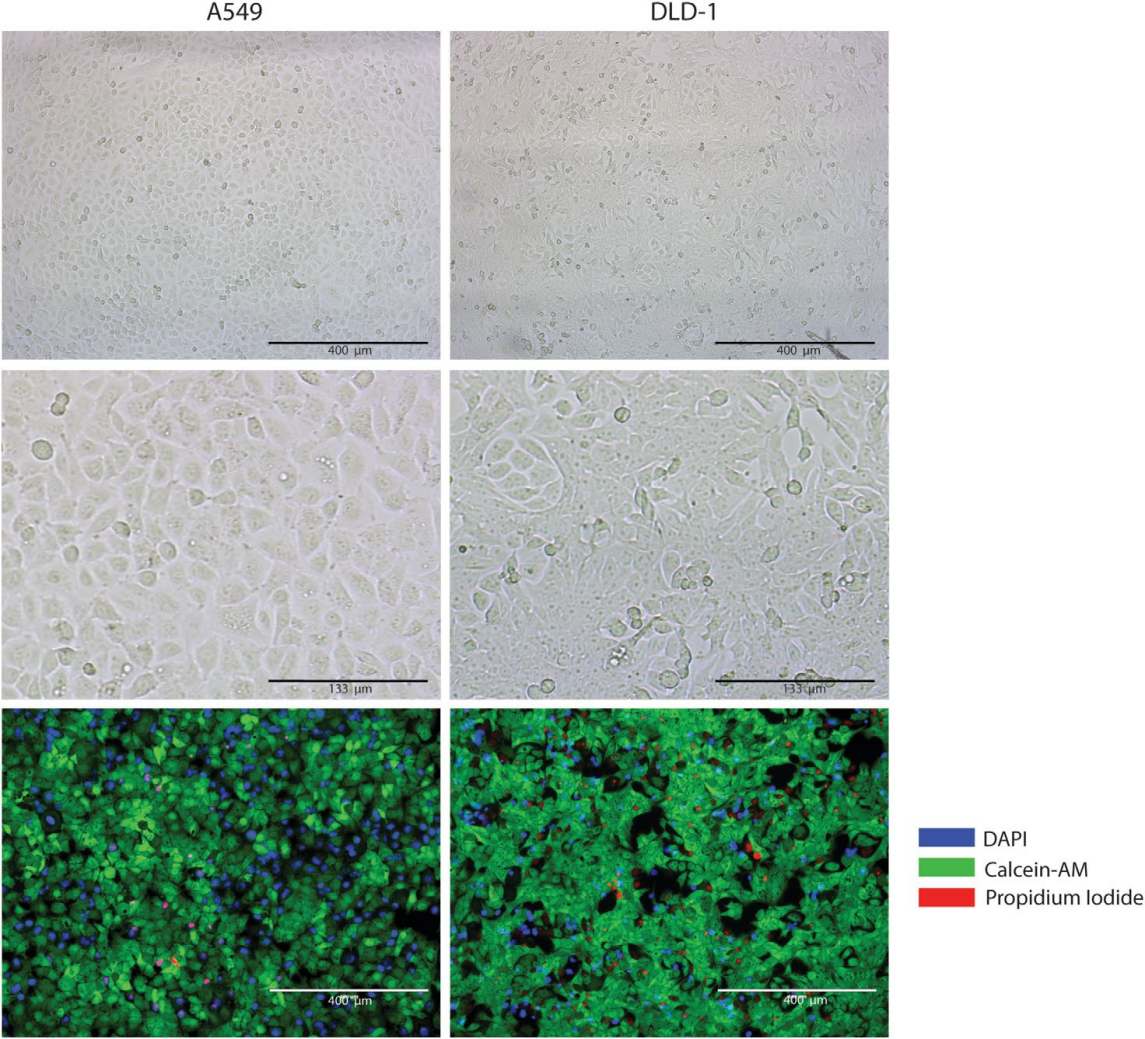
Connection of A549 cells to DLD‐1 cells does not induce cell death or morphological abnormalities. Lung epithelial A549 cells and colorectal epithelial DLD‐1 cells were grown in the multi‐organ‐on‐chip (MOoC) system, in separate culture chambers, under 150 μL/h of constant medium flow. Upon reaching confluency, the A549 cells were connected to DLD‐1 cells for 24 h. Medium, including all secreted factors from A549 cells, constantly stimulated the DLD‐1 cells. Afterwards, cells were visually inspected using light microscopy as well as examined for loss of cell viability using a fluorescent live dead staining, consisting of Calcein‐AM (viable cells; green), Propidium Iodide (dead cells; red) and Hoechst (nuclei; blue). Representative images of three independent experiments.

In order to study interorgan communication by circulating mediators between different cell types, a novel Multi‐Organ‐on‐Chip (MOoC) system was developed (Figure 1). The MOoC system consists of two 100 mm^2^ cell culture chambers that can either be used separately or be serially connected to a syringe pump to generate a millifluidic medium flow. Upon connection of the two cell culture chambers, any secreted factors will travel with a flow rate of 150 μL/h from the first to the second cell culture chamber, subsequently exposing cells in the second cell culture chamber. To study communication from lung cells to colorectal cells, submerged lung epithelial A549 cells were cultured to a monolayer in the first cell culture chamber, and colorectal epithelial DLD‐1 cells were cultured in the second cell culture chamber. After the cell culture chambers were connected for 24 h, the cells were microscopically inspected for morphological changes and for loss of viability using a live/dead staining (Figure 3). No morphological changes or increased rates of cell death were observed upon connection of the cells, indicating that DLD‐1 cells tolerate exposure to secreted factors from non‐stimulated A549 cells.

### 3.2 Nylon Microplastic Fibers Induce the Release of Interorgan Communication Mediators

**FIGURE 4:**
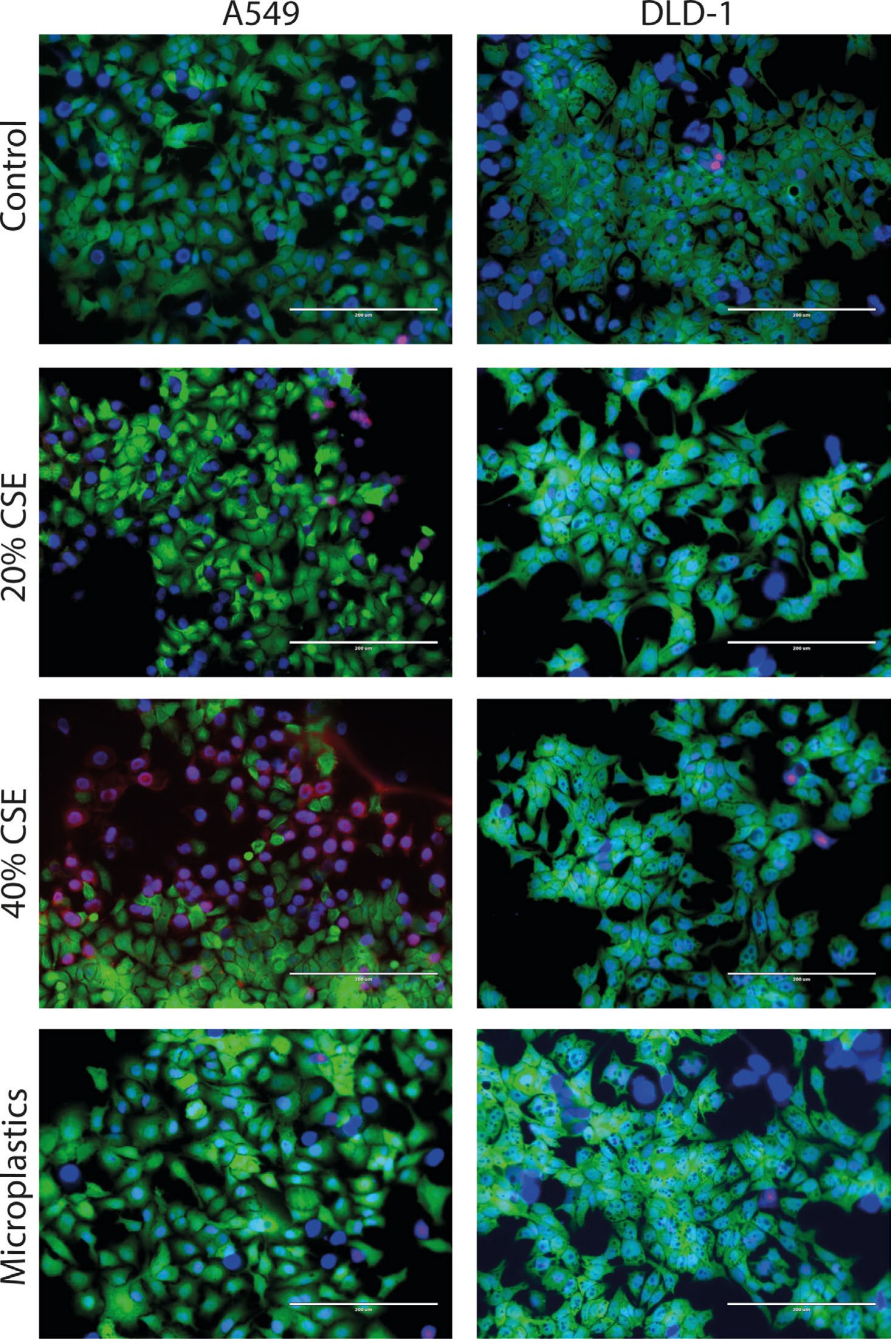
Cell viability upon connection of A549 cells exposed to cigarette smoke extract (CSE) or microplastics to DLD‐1 cells. Lung epithelial A549 cells were grown in the multi‐organ‐on‐chip (MOoC) system, under 150 μL/h of constant medium flow, until reaching confluency. Subsequently, A549 cells were stimulated with the inhalable pollutants, 20% cigarette smoke extract (CSE), 40% CSE and 10,000 nylon microplastic fibers for 4 h, followed by extensive flushing to remove the stimuli, followed by 24 h of incubation under flow. Afterwards cells were examined for loss of cell viability using a fluorescent live dead staining, consisting of Calcein‐AM (viable cells; green), Propidium Iodide (dead cells; magenta) and Hoechst (nuclei; blue). Representative images of three independent experiments.

**FIGURE 5:**
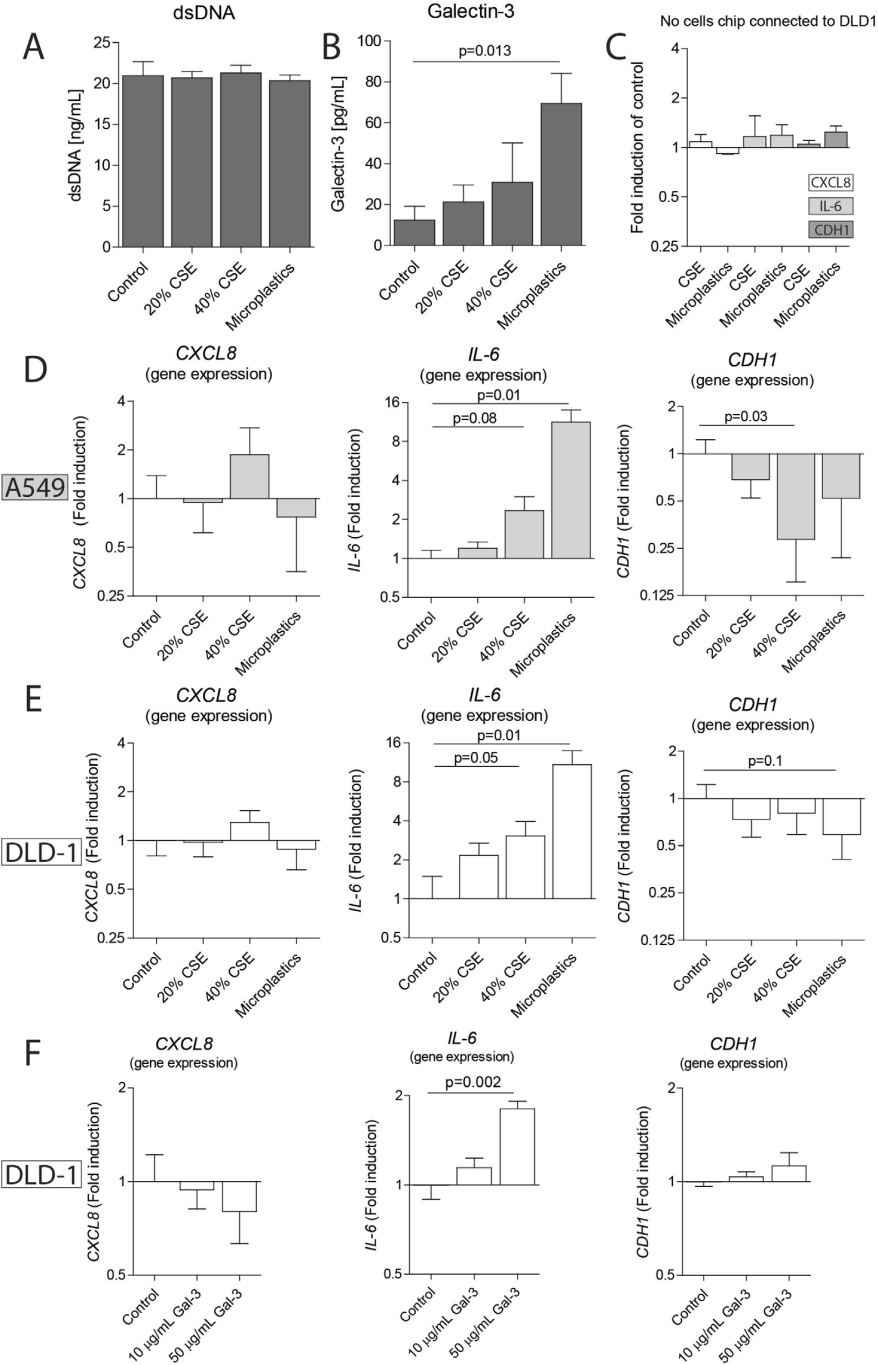
Pollutant‐exposed lung cells activate colorectal cells by communication via released factors. Lung epithelial A549 cells and colorectal epithelial DLD‐1 cells were grown in the multi‐organ‐on‐chip (MOoC) system, under 150 μL/h of constant medium flow, until reaching confluency. Subsequently, A549 cells were stimulated with the inhalable pollutants, 20% cigarette smoke extract (CSE), 40% CSE and 10,000 nylon microplastic fibers for 4 h, followed by extensive flushing to remove the stimuli. Next, the A549 cells were connected to DLD‐1 cells for 24 h. Levels of the interorgan communication mediators (A) dsDNA and (B) galectin‐3 were measured in chip outflow. In order to identify whether factors of CSE or microplastics can adhere to the chip and affect DLD‐1 cells without the involvement of A549 cells, a chip containing DLD‐1 cells was connected to a chip without cells. The empty chip was stimulated with 40% CSE or 10,000 nylon microplastic fibers for 4 h, followed by extensive flushing to remove the stimuli. (C) The DLD‐1 gene expression levels of *CXCL8*, *IL‐6* and *CDH1* were shown as fold induction of 40% CSE or microplastic‐stimulated chips compared to the unstimulated empty chip. Upon connection of A549 cells to DLD‐1 cells, the gene expression of the pro‐inflammatory cytokines *CXCL8* and *IL‐6* and the epithelial barrier gene *CDH1* were measured in (D) A549 cells and (E) DLD‐1 cells. (F) DLD‐1 cells were stimulated in the chip with 0, 10, 50 μg/mL recombinant human Galectin‐3 for 24 h. Afterwards, the gene expression levels of *CXCL‐8*, *IL‐6* and *CDH1* were measured. Gene expression shown as fold induction relative to the non‐stimulated control. All experiments performed in *n* = 6–7, and all data is shown as mean ± SEM. Significance was tested using a Mann–Whitney *U* test, *p* values are indicated on a line between the tested conditions.

To study whether exposure of lung epithelial cells to cigarette smoke and microplastics leads to the release of long‐lived soluble factors that affect colorectal cells, A549 cells were exposed to cigarette smoke extract (20%–40%) or 10,000 nylon microplastic fibers in the MOoC system (Figure 2). Extensive flushing after a 4‐h stimulation period of the A549 cells was performed to remove all CSE or microplastic fibers. Next, the cell lines were connected in the MOoC system for 24 h. Afterwards, the effects on cell viability were investigated by performing a live‐dead staining in the chips. Exposure of A549 cells to 20% CSE or microplastics did not induce cell death (Figure 4). However, exposure of A549 cells to 40% CSE did increase the number of dead cells. The connection of CSE‐ or microplastic‐exposed A549 cells to DLD‐1 cells did not induce cell death in DLD‐1. Next, the levels of dsDNA were assessed in the outflow collected after passing through both cell culture chambers. No increase in the levels of dsDNA was observed when comparing the outflow of DLD‐1 cells connected to CSE/microplastic‐stimulated A549 cells to DLD‐1 cells connected to non‐stimulated A549 cells, indicating that neither CSE nor microplastic exposure induced significant levels of necrotic cell death‐related DAMP release (Figure 5A). Next, levels of the pro‐inflammatory interorgan communication mediator galectin‐3 were measured in the outflow. No significant increase of galectin‐3 was observed in the outflow of DLD‐1 cells connected to CSE‐stimulated A549 cells. However, levels of galectin‐3 were significantly higher in the outflow of DLD‐1 cells connected to microplastic‐stimulated A549 cells compared to the outflow of DLD‐1 cells connected to non‐stimulated A549 cells (Figure 5B). These results indicate that exposure of lung epithelial cells to microplastics triggers the release of soluble communication mediators.

### 3.3 Inhalable Pollutant‐Exposed A549 Cells Induce a Pro‐Inflammatory Response in DLD‐1 Cells

As it was not possible to discriminate between the release of secreted factors from A549 and DLD‐1 cells in the chip outflow using our experimental set‐up, we next measured the effects of exposing lung cells to inhalable pollutants on the expression of pro‐inflammatory and epithelial barrier genes in colorectal cells using qPCR. Hereto, gene expression of *IL‐6*, *CXCL8*, and *CDH1* (encoding the barrier protein E‐cadherin) was analyzed in A549 and DLD‐1 cells separately. Firstly, a control experiment was performed to identify whether any potential residual factors of CSE or microplastics can directly affect DLD‐1 cells. Hereto, a chip containing DLD‐1 cells was connected to a chip without any cells that was pre‐treated with CSE or microplastics. The gene expression levels of *CXCL‐8*, *IL‐6*, or *CDH1* did not significantly change in DLD‐1 cells upon connection to pre‐exposed chips without cells (Figure 5C). Next, A549 cells were exposed to CSE or microplastics in the chip. Exposure of A549 cells to CSE or microplastics did not significantly affect *CXCL8* expression (Figure 5D). However, a trend towards higher gene expression of *IL‐6* was observed upon exposure of A549 cells to 40% CSE, while the expression of IL‐6 was significantly higher upon exposure to nylon microplastics. Additionally, gene expression levels of *CDH1* were lower upon exposure of A549 cells to 40% CSE compared to the non‐exposed A549 cells.

Next, a chip containing DLD‐1 cells was connected to a chip containing pre‐treated A549 cells. The expression of IL‐6 was increased in DLD‐1 upon connection to CSE‐stimulated A549 cells compared to DLD‐1 cells connected to non‐stimulated A549 cells (Figure 5E). Connection of DLD‐1 cells to microplastic‐stimulated A549 cells induced higher *IL‐6* expression as well as a trend towards lower *CDH1* expression. As DLD‐1 cells were not exposed to CSE or microplastics themselves, these results indicate that the exposure of lung epithelial cells to inhalable pollutants triggers the release of soluble factors that induce a pro‐inflammatory response and reduce barrier gene expression in colorectal cells.

To further study whether these effects in DLD‐1 cells could have been induced by factors secreted from A549 cells, DLD‐1 cells were stimulated in the chip with recombinant human Galectin‐3. In agreement with the inter‐organ communication experiments, an increase in the expression of *IL‐6* was observed, but no change in *CXCL8* or *CDH1* expression (Figure 5F).

### 3.4 Exposure of Lung Epithelial Cells to Inhalable Pollutants Decreases the Barrier Integrity of Colorectal Cells

**FIGURE 6:**
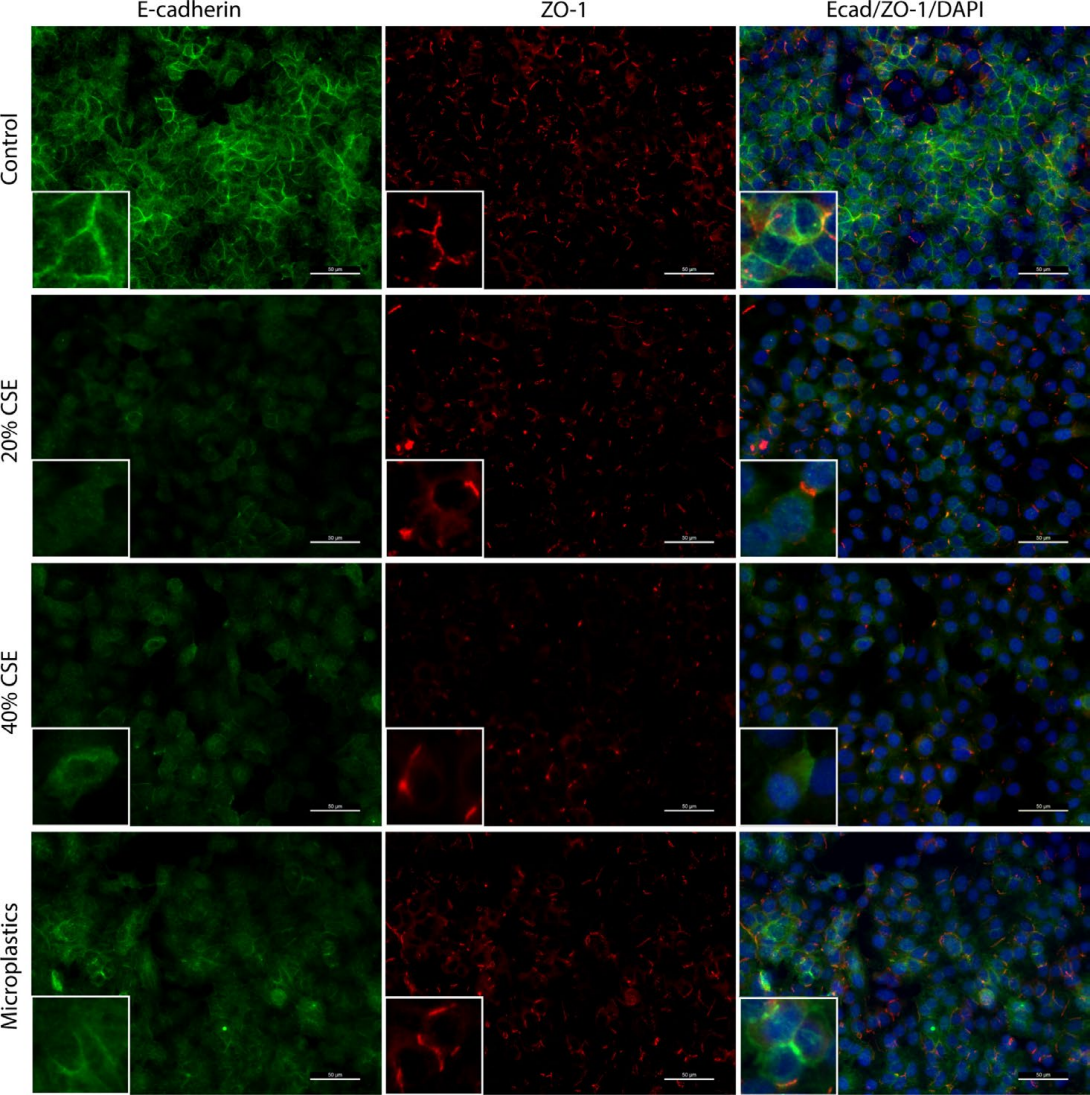
Exposure of A549 cells to cigarette smoke extract (CSE) or microplastics disrupts epithelial barrier integrity. Lung epithelial A549 cells were grown in the multi‐organ‐on‐chip (MOoC) system, under 150 μL/h of constant medium flow, until reaching confluency. Subsequently, A549 cells were stimulated with the inhalable pollutants, 20% cigarette smoke extract (CSE), 40% CSE and 10,000 nylon microplastic fibers for 4 h, followed by extensive flushing to remove the stimuli, followed by 24 h of incubation under flow. Afterwards the barrier integrity was assessed by performing a fluorescent staining for E‐cadherin (green), Zonula Occludens‐1 (ZO‐1; red) and nuclei (DAPI; blue). The white ruler indicate 50 μm on the main picture and 150 μm on the zoomed‐in picture (bottom left corner of each picture). Presented are representative images of 3 independent experiments.

In order to further investigate epithelial barrier integrity, we assessed whether the reduction in *CDH1* expression upon the connection of colorectal cells to inhalable pollutant‐exposed lung cells was accompanied by delocalization of the adherens and tight junction proteins E‐cadherin and ZO‐1 in colorectal cells. In non‐exposed A549 cells, a clear and localized membrane staining pattern was visible for E‐cadherin, as well as a slightly scattered but membrane‐localized signal for ZO‐1 (Figure 6). Stimulation of A549 cells with CSE, and to a lesser extent microplastics, induced a disrupted expression pattern of both E‐cadherin and ZO‐1. For E‐cadherin, the expression was decreased in both the cytoplasm as well as the cell membrane by both CSE and microplastics, while for ZO‐1, the expression was specifically altered on the cell membrane (Figure 8A). Forty percent CSE decreased the expression of ZO‐1, while microplastics increased the expression of ZO‐1 on the cell membrane.

**FIGURE 7:**
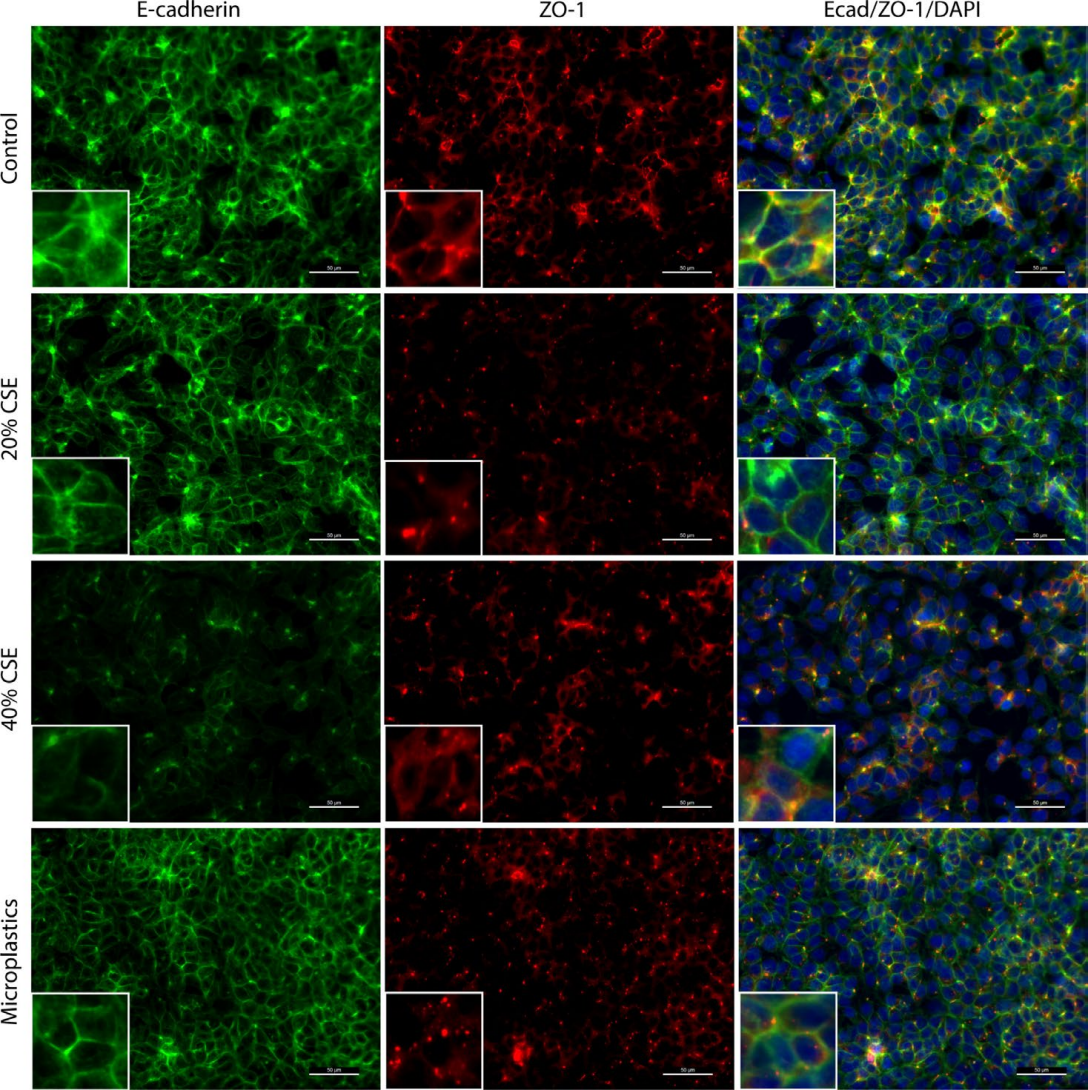
Cigarette smoke extract (CSE)‐exposed A549 cells decrease the barrier integrity of colorectal DLD‐1 cells via interorgan communication. Lung epithelial A549 cells and colorectal epithelial DLD‐1 cells were grown in the multi‐organ‐on‐chip (MOoC) system, under 150 μL/h of constant medium flow, until reaching confluency. Subsequently, A549 cells were stimulated with the inhalable pollutants, 20% CSE, 40% CSE and 10,000 nylon microplastic fibers for 4 h, followed by extensive flushing to remove the stimuli. Next, the A549 cells were connected to DLD‐1 cells for 24 h. Afterwards the barrier integrity was assessed in the DLD‐1 cells by performing a fluorescent staining for E‐cadherin (green), Zonula Occludens‐1 (ZO‐1; red) and nuclei (DAPI; blue). The white ruler indicate 50 μm on the main picture and 150 μm on the zoomed‐in picture (bottom left corner of each picture). Presented are representative images of 3 independent experiments.

**FIGURE 8:**
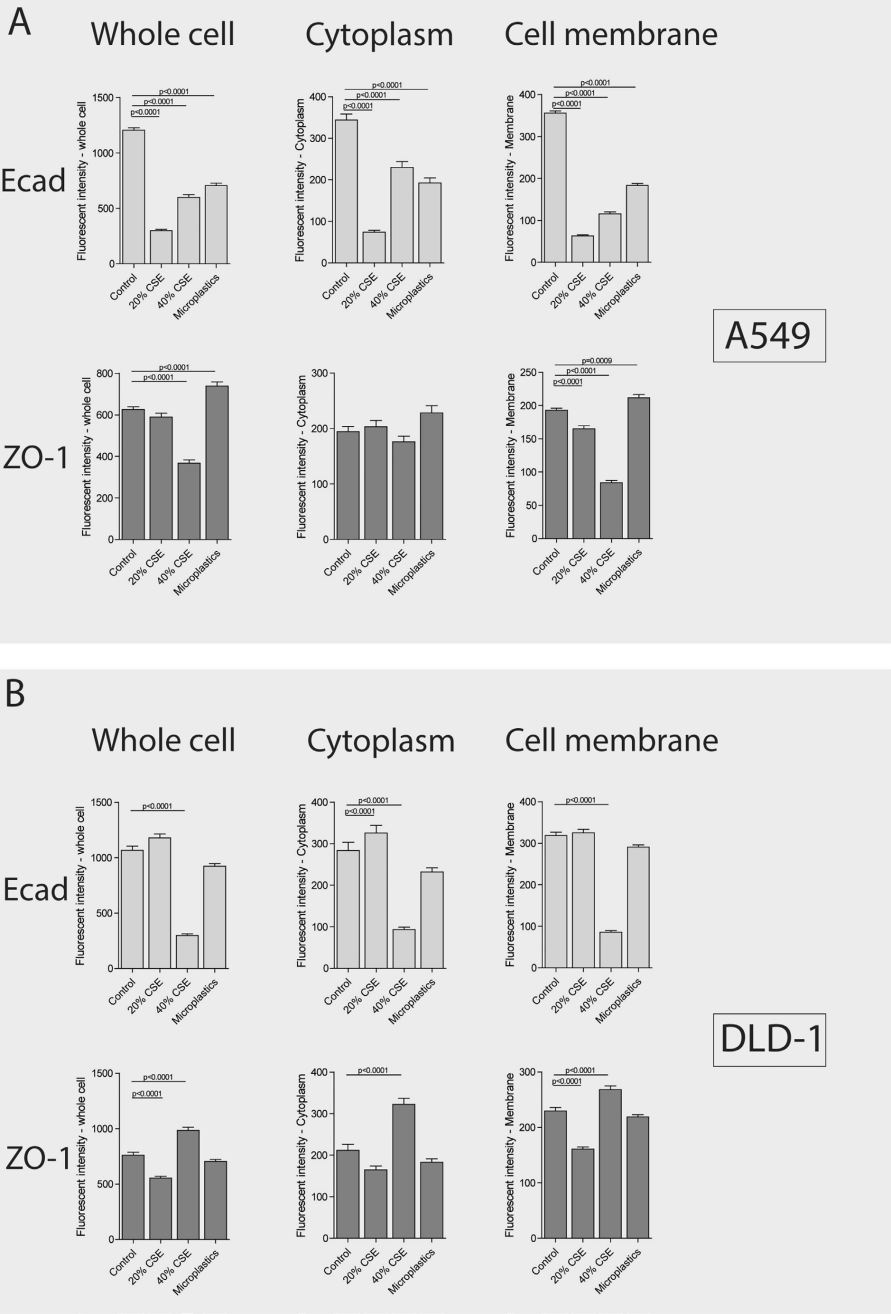
E‐cadherin and Zonula Occludens‐1 localization in A549 and DLD‐1 cells. (A) The expression of E‐cadherin (Ecad) and Zonula Occludens‐1 (ZO‐1) in A549 cells upon exposure to 20% or 20% cigarette smoke extract (CSE) or 10,000 nylon microplastic fibers (MP), grown in the multi‐organ‐on‐chip (MOoC) system, under 150 μL/h of constant medium flow. The fluorescent intensity (FI) of the Ecad and ZO‐1 expression was assessed in 100–400 individual cells in the whole cell, the cytoplasm (whole cell minus nucleus and cell membrane) and the cell membrane. (B) The expression of Ecad and ZO‐1 in DLD‐1 upon connection to CSE (20%–40%) or MP‐exposed A549 cells. The DLD‐1 cells were connected to A549 cells in the MOoC system under 150 μL/h of constant medium flow for 24 h. The FI of the Ecad and ZO‐1 expression was assessed in 200–400 individual cells in the whole cell, the cytoplasm (whole cell minus nucleus and cell membrane) and the cell membrane. Ecad expression is shown in light gray bars, ZO‐1 expression is shown as dark gray bars. Data is shown as mean FI ± SEM. Significance was tested using a Mann–Whitney *U* test, *p* values are indicated on a line between the tested conditions.

Similar to in A549 cells, clear membrane‐localized staining was visible for both E‐cadherin as well as ZO‐1 in DLD‐1 cells that were connected to non‐stimulated A549 cells (Figure 7). Connection of DLD‐1 cells to A549 cells stimulated with 40% CSE and to a lesser extent microplastics altered the expression of Ecad and ZO‐1 in DLD‐1 cells. The Ecad expression was lower in the cytoplasm as well as the cell membrane upon connection to A549 cells exposed to 40% CSE and to a lesser extent microplastics (Figure 8B), while the ZO‐1 expression was increased in the cytoplasm as well as the cell membrane upon connection to A549 cells exposed to 40% CSE. Together, these results confirm that exposure of lung epithelial cells to cigarette smoke or microplastics not only affects the lung epithelial barrier integrity, but also triggers the release of soluble factors that can disrupt the barrier integrity of colorectal cells.

## 4 Discussion

The current study presents a novel millifluidic multi‐organ‐on‐chip system suitable for studying interorgan communication via soluble circulating mediators. We used this model to assess whether exposure of lung epithelial cells to CSE or microplastic fibers can lead to the transfer of signals that affect epithelial cells of the gut. Our study showed that lung cells exposed to inhalable pollutants can communicate with colorectal cells, inducing pro‐inflammatory responses and reducing the barrier integrity of these cells.

In our experimental set‐up, exposure of lung epithelial cells to CSE or microplastics induced a pro‐inflammatory response and a decrease in barrier integrity. This is in agreement with previous studies (Ferraro et al. 2023; Kode et al. 2006). The effects of microplastics on A549 cells are less well described and less consistent. Although chemically different, it was shown that polystyrene nanoplastics also induce a pro‐inflammatory response upon exposure of A549 cells (Milillo et al. 2024). However, another study reports complete non‐responsiveness of A549 cells upon stimulation with polystyrene nanoplastics (Gosselink et al. 2024). In our study, exposure of A549 cells to nylon microplastic fibers induced a four‐fold increase in galectin‐3 release. Galectin‐3 is a well‐known interorgan communication mediator that upon release from cells can induce a pro‐inflammatory response. Galectin‐3 is expressed in various immune cells, but also in other cell types, like endothelial and epithelial cells (de Oliveira et al. 2015). Galectin‐3 can be passively secreted upon cell death, acting as a DAMP, but can also be actively secreted upon cellular stress or upon exposure to stimuli like lipopolysaccharide (LPS) or interferon‐γ (Díaz‐Alvarez and Ortega 2017). When galectin‐3 is released into the extracellular space, it can exert pro‐inflammatory responses and activate several cell types, including monocytes, lymphocytes, dendritic cells, neutrophils, and airway epithelial cells (Díaz‐Alvarez and Ortega 2017; Gilson et al. 2019). Previously, we have shown that exposure of airway epithelial cells to CSE induces galectin‐3 release in a dose‐dependent manner, and that this response is exaggerated in COPD patients (Pouwels et al. 2016). Additionally, we have shown that stimulation of COPD‐derived airway epithelial cells with recombinant galectin‐3 induces Toll‐Like Receptor (TLR)‐2/4‐dependent CXCL8 release (Pouwels et al. 2016). This is in agreement with other studies showing that cigarette smoking induces an increase in expression and release of galectin‐3 (Sharma et al. 2024; Sundqvist et al. 2021). The results from our study are also in line with a recent study showing that stimulation of vascular smooth muscle cells with polyethylene and polystyrene microplastics led to an increase in the expression of galectin‐3 (Persiani et al. 2025). Although no increase in *CXCL8* mRNA expression was observed in A549 cells upon exposure to CSE or microplastics, we did observe an increase in *IL‐6* expression. It is possible that the released galectin‐3 induced the increase in *IL‐6* expression. Subsequently, the lung cell‐derived IL‐6 can also contribute to the observed pro‐inflammatory effects in colorectal cells.

In order to study interorgan communication, lung cells exposed to CSE or microplastic fibers were connected to naïve colorectal cells within our MOoC system. Extensive flushing of the cell culture chips ensured that the colorectal cells were never exposed to CSE or microplastic fibers, but were only stimulated with factors released from the lung epithelial cells. The constant flowrate of 150 μL per hour was previously shown to induce minimal levels of shear stress not affecting cellular behavior, (Rae et al. 2025) yet ensuring a constant and equal exposure to released factors. The colorectal cells responded to the released factors from lung epithelial cells by the induction of a pro‐inflammatory response, as evidenced by increased *IL‐6* expression and loss of their barrier integrity, demonstrated by delocalized E‐cadherin and ZO‐1 expression. A reduction in gut barrier integrity and induction of intestinal inflammation was previously shown to occur upon cigarette smoke exposure in a rat and a mouse model, (Xin et al. 2016; Zuo et al. 2014) indicative of the activated bi‐directional gut‐lung‐axis. Similar effects on intestinal health have been observed in smoking COPD patients (Wang et al. 2023).

In our study, it was shown that stimulation of A549 cells to CSE and especially nylon microplastic fibers induced the release of the DAMP Galectin‐3. Additionally, we showed that stimulation of DLD‐1 cells with recombinant human Galectin‐3 increased the expression of *IL‐6*, which was in agreement with the effects seen upon connection of DLD‐1 cells to CSE or microplastic‐stimulated A549 cells. Nevertheless, it is likely that Galectin‐3 is not solely responsible for the inter‐organ communication between lung and colorectal cells, and that a variety of other factors also contribute to the observed inter‐organ communication effects.

Several studies have shown that the levels of various interorgan communication mediators, such as cytokines, chemokines, DAMPs, and extracellular vesicles, are increased in the circulation of COPD patients (Pouwels et al. 2014; Pouwels et al. 2015; Petraroia et al. 2023; Bade et al. 2014). However, to date, limited functional studies were performed on the role of interorgan communication mediators in COPD. To the best of our knowledge, only one study used pulmonary extracellular vesicles to stimulate cardiomyocytes, which affected cardiomyocyte functioning and viability (Liu et al. 2024). This limited availability of data on interorgan communication in COPD may be due to a lack of suitable in vitro models to effectively study the factors and mechanisms involved in the communication between cells derived from different organs. Classical cell co‐culture models typically use one or two cell types derived from the same organ in a static system. In vitro cell culture models are developing rapidly, including the development of organ‐on‐chip models. Recently developed lung‐on‐chip models include a stretchable lung‐on‐chip model, a large‐scale millifluidic airway‐on‐chip model, a microvessel‐on‐chip model, and a breathing mucociliary‐on‐chip platform (Rae et al. 2025; Pandian et al. 2025; Richter et al. 2025; Lin et al. 2025). Additionally, other multi‐organ‐on‐chip models have been developed that incorporated a lung compartment (Xu et al. 2016; Skardal et al. 2017). However, to the best of our knowledge, these models have not left the developmental phase and have not been used to study the communication between cells derived from different organs. The effective usage of recently developed and commercially available multi‐organ‐on‐chip platforms still poses challenges, including high set‐up and running costs, suitability for culturing lung‐derived epithelial cells, and obtaining biological samples large enough for standard readout technologies, including qPCR and ELISA. To address these issues, we have developed an open‐source large‐scale airway‐on‐chip system, which is cost effective, is easy to implement in any lab environment, and has a large 100 mm^2^ cell culture area, comparable with standard 24‐well inserts used for conventional air‐liquid interface culturing of airway epithelial cells (Rae et al. 2025). In the current study, we have further developed this airway‐on‐chip model to function as a millifluidic multi‐organ‐on‐chip model suitable for studying interorgan communication by released circulating factors.

The response of cells towards released factors from other cells can also be studied with simpler in vitro models, such as co‐culture models or conditioned medium transfer models (Yang et al. 2024; Pramil et al. 2021). However, with co‐culture models the direction of communication can not be studied as secreted factors travel freely between cell types. When transferring conditioned medium, a large amount of released factors will be applied to cells at once, which can induce a different response compared to a slow and continuous exposure, while short‐lived factors may have already disappeared. In our MOoC model, a constant flow of 150 μL per hour ensures the constant and stable stimulation of cells with released factors, immediately upon release from a secondary cell type.

In summary, we developed an open source MOoC model that can be set up with limited means and can easily be adjusted to encompass different cell types, stimuli, and read‐out systems. A unique feature of our MOoC system is that it has two large 100 mm^2^ cell culture areas, yielding large quantities of biological samples suitable for a plethora of biological assays. In the future, this model can be utilized for various studies investigating the communication between different cell types by released factors.

In the current study, the MOoC system was used to study the communication between lung epithelial cells stimulated with the inhalable pollutants CSE and nylon microplastic fibers, and naïve colorectal epithelial cells. We show that factors released from pollutant‐exposed lung epithelial cells induce inflammation and reduce barrier integrity of colorectal epithelial cells, characteristics that are also often observed in COPD patients. This study may provide important information on the mechanisms of the lung–gut axis in COPD, potentially driving the onset of gastrointestinal COPD co‐morbidities.

## Author Contributions

S.D.P., I.H.H., D.‐J.S., J.K.B., and A.N. conceived and designed research. S.D.P., B.R., V.B., and H.‐J.D. performed experiments. S.D.P., V.B., and H.‐J.D. analyzed data. G.F.V. and B.N.M. supplied standardized nylon fibers. S.D.P. interpreted the results of experiments. S.D.P. and H.‐J.D. prepared figures. S.D.P. drafted the manuscript. I.H.H., B.R., D.‐J.S., J.K.B., A.N., B.N.M., and G.F.V. edited and revised the manuscript. B.R., V.B., H.‐J.D., G.F.V., B.N.M., A.N., J.K.B., D.‐J.S., I.H.H., and S.D.P. approved the final version of themanuscript.

## Conflicts of Interest

The authors declare no conflicts of interest.

## Data Availability

All data is shown within this manuscript. Raw data and 3D‐print files are available upon reasonable request to the corresponding author.
